# The Wisconsin Dental College

**Published:** 1884-05

**Authors:** Adolf Petermann

**Affiliations:** Frankfort-on-the-Main


### THE WISCONSIN DENTAL COLLEGE.

*Translated for the Independent Practitioner from a Paper*

BY DR. ADOLF PETERMANN, FRANKFORT-ON-THE-MAIN.

*In Deutsche Monatsschrift fuer Zahnheilkunde for March,* 1884.

It is but a very few years ago that the trade in doctors' diplomas, which mainly originated from the "doctor factory" of *John Buchanan* in Philadelphia, was stopped through the unmasking of Mr.

Buchanan, and sending him to prison. Again, there are signs of a revival in this ignominious business. At that time as well as at present, it was principally in Europe where their sale was attempted. Before the evil has grown again to larger dimensions it will be well to present the following by way of enlightenment:

The *New-Yorker Staatszeitung*, Number 62, of March 14th, 1881, says: "Wisconsin also rejoices in the possession of a doctor-diploma factory, though it is for dentistry only. The dental college in Delavan (Wisconsin Dental College) furnishes such diplomas *in absentia*, for $12. The whole college consists of a few poorly furnished rooms in a second story, and its president, Mr. Geo. Morrison, calling himself doctor, is at once secretary, treasurer, and, very likely, the whole college in person. However, besides Morrison, there are also engaged two professors, namely John Morrison (son of the president), professor of dental surgery, and Dr. D. B. Devendorf, professor of anatomy and surgery."

In the advertising columns of the *Kladderadatsch*, the *Fliegenden Blatter*, etc., could be seen lately an advertisement offering the "dental diploma of an American University." Upon asking for further information the always ready agent, who is domiciled in England, furnished the following answer:

"West Grimstead, April 17th, 1883.

"Dear Sir.—In reply to your communication I wish to say that I can procure you the diploma of Doctor of Dentistry from a Dental Academy in the State of Wisconsin, America. The condition is the filing of a paper pertaining to dentistry or dental practice. In America, whoever is a dentist is a doctor, but if desired, the additional insertion that you were created a dentist may be made. The transaction of course to be confidential. Cost, 500 marks. After you have made up your mind about getting the degree, I shall be pleased to give further information and attend to the matter. The academy is authorized by the State to confer the degree of doctor, and the diplomas are recognized everywhere in the United States.

"Respectfully,

"W. Rolt.

"West Grimstead, Sussex, England."

Last year the fraudulent concern called the "Wisconsin Dental College" sent its offers *directly* to all conceivable addresses in Germany. This fine college offers its diplomas for doctor of dental surgery for $12 cash. In order to disguise the enormous swindle, these marketable and worthless diplomas are conferred *honoris causa*, for, as the inquiries instigated by the German Imperial Chancellor have shown, and as had been confirmed by the American Minister in Berlin, American Universities and Colleges are not allowed to confer degrees *in absentia.**

We now see the American law evaded through the addition of *honoris causa.*

Enclosed in one of these offers was the following order in blank for such a diploma:

..............................188 .

This statement is made to the Faculty of the Wisconsin Dental College, located at Delavan, Walworth Co., Wisconsin, for the purpose of procuring an honorary diploma and degree D. D. S. (Doctor of Dental Surgery).

I am a regular practicing dentist.

I am a resident of ..........................................

Age........years. Have practiced dentistry..............years.

Signed............................

Witness..................

July 2d, 1882, the courts in Bremen sentenced, in due form, the dental artificer, Edward Hanft, to a fine of sixty marks, or twelve days' imprisonment, for illegally using the title of doctor, and for assuming additionally the title of American dentist; they also ordered the removal of his sign, and of his so-called dental diploma. Hanft declared that, based upon a diploma of the Wisconsin Dental College, he considered himself justified in using the above titles. It was established in the judicial proceedings that this college is a swindling corporation, which has but the single aim of selling dental diplomas. The German Consul in Chicago reported concerning the Wisconsin Dental College as follows: "Unconditional liberty of

*See *Zahnarztlicher Almanach* (Johannes Alt, Frankfort-on-the-Main, publisher). Vol. 1879, page 86, and vol. 1881, page 144.

trade is ruling there. Two or three persons may form a corporation, and acquire for it the rights of a judicial person. The president of the college, Mr. Morrison, told him in reference to the accused, Hanft, that he had been extraordinarily well recommended to him (Morrison) and his colleagues. Hanft informed him (Morrison) that he had practiced as a dentist in Europe for five years, whereupon the degree of doctor was conferred upon him."

The Attorney-General proved that Hanft did not occupy himself during five years with diseased teeth, but with butter instead, in the store of a dealer in Hanover. Thus, based upon untrue statements and without preceding examination, he procured, for a money consideration his dental diploma, which was devoid of any value.

The Secretary of the Interior at Washington, and the Postmaster General of the United States of North America, have issued the following letter in reference to the fraudulent transactions of the Wisconsin Dental College:

POSTOFFICE DEPARTMENT,
WASHINGTON, D. C., July 13, 1881.

*The Honorable the Secretary of the Interior.*

SIR.—I have the honor to acknowledge the receipt of your communication of the 11th instant, referring to this department a communication from the Commissioner of Education, enclosing letters, etc., showing the fraudulent character of business carried on by one Dr. GEORGE MORRISON, President of Wisconsin Dental College, at Delavan, Wis.

In reply I have to state that I have this day issued an order to the Postmaster at Delavan, Wis., forbidding the payment of money orders, or the delivery of registered letters, to the said Dr. GEORGE MORRISON.

Very respectfully,
THOMAS L. JAMES,
Postmaster-General.

But, besides the above communications, I am assured that the Wisconsin Dental College, with its nefarious trade in dental diplomas, has many times been exposed by American dental periodicals. Noticeably by the Dental Cosmos (Chestnut Street, corner of

12th, Philadelphia), vol. 1881, pages 157, 490, 555, and vol. 1884, page 28. Also, Johnston's Dental Miscellany (1260 Broadway, New York) speaks very elaborately on the fraudulent and shameless operations of Mr. Morrison and his so-called Wisconsin dental college.

---

### Second Article from the same Journal, of April, 1884.

*Translated for the Independent Practitioner.*

BY MISS CORA FREEMAN.

To my communications in the previous number of this Journal, I to-day add the following:

The Frankfort newspaper published in its advertising columns the following announcement: "Doctors' diplomas of dentistry, philosophy, law, etc., are furnished honorably and secretly. Address, C. R. (care of Stationer), 10 Duke Street, Bloomsbury, London, W. C."

In answer to inquiries made at the above-mentioned place, under an assumed name, two letters were received from a certain "Professor" G. Rumler. The first letter is not especially noteworthy. It is an ordinary general offer in which secrecy is promised. The second letter reads as follows:

London, 32 Thornhill Crescent, Barnsbury.
March 10, 1884.

*Dear Sir:*

In answer to your honored letter, I respectfully inform you that you are negotiating with a *gentleman*, who procures for you the diploma of a thoroughly respectable Institute, and one that is entitled to confer the degree of doctor. The name of the institution in German is "Wisconsiner Zahnarztliche Academie" (Wisconsin Dental College).

The fee you can deposit in any banking house in Frankfort, and have the deposit in these negotiations credited to me, upon which I will immediately order the diploma for you. If you do not wish to enter into any bank business on trust, the simplest way would be for you to send me two hundred marks now, and the remaining two hundred marks on receipt of the diploma. With the exception of the four hundred marks, you have no further costs. Please send your full name with order.

Further, I beg you to subscribe your name to the enclosed printed offer of promotion, at the placc where the word "signed" is printed.* The rest I will fill out myself. You see on the back of this circular the statement of the president of the college, that the degree of doctor can only be conferred after it has been confirmed by me. It will be about five weeks before I can furnish you with the diploma.

While I respectfully await news from you, as well as the return of the subscribed order, I am,

Yours very truly,

PROFESSOR DR. G. RUMLER.

The words written on the back of the aforementioned circular read as follows:

DELAVAN, WIS., U. S. A.

This statement will be honored from any regular practicing dentist, if endorsed by Prof. Dr. Rumler.

GEO. MORRISON, President.

So this *gentleman*, Mr. "Professor Dr." Rumler, procures for everybody (even under an assumed name), without any other consideration, on payment of 400 marks, and without any scientific qualifications or examination of circumstances, a diploma as doctor from the *respectable* Wisconsin Dental College. A fine state of affairs. From my first article on this college it can be learned that Mr. Morrison, for only $12, furnished a similar diploma, for which 400 M. are demanded here. Truly, Mr. Rumler carries on a disreputable but profitable trade.

*Such a document is printed in the preceding communication, but the new one has the addition of a raised stamp, " Wisconsin Dental College, Incorporated July, 17, 1880." P.

